# Incidence and prevalence of dementia in linked administrative health data in Saskatchewan, Canada: a retrospective cohort study

**DOI:** 10.1186/s12877-015-0075-3

**Published:** 2015-07-03

**Authors:** Julie G. Kosteniuk, Debra G. Morgan, Megan E. O’Connell, Andrew Kirk, Margaret Crossley, Gary F. Teare, Norma J. Stewart, Vanina Dal Bello-Haas, Dorothy A. Forbes, Anthea Innes, Jacqueline M. Quail

**Affiliations:** Canadian Centre for Health and Safety in Agriculture, University of Saskatchewan, PO Box 23, 104 Clinic Place, Saskatoon, S7N 2Z4 SK Canada; Canadian Centre for Health and Safety in Agriculture, University of Saskatchewan, Saskatoon, SK Canada; Department of Psychology, University of Saskatchewan, Saskatoon, SK Canada; Division of Neurology, College of Medicine, University of Saskatchewan, Saskatoon, SK Canada; Department of Psychology (Professor Emerita), University of Saskatchewan, Saskatoon, SK Canada; Saskatchewan Health Quality Council, Saskatoon, SK Canada; College of Nursing, University of Saskatchewan, Saskatoon, SK Canada; School of Rehabilitation Science, McMaster University, Hamilton, ON Canada; Faculty of Nursing, University of Alberta, Edmonton, AB Canada; Bournemouth University Dementia Institute, Bournemouth University, Dorset, UK

**Keywords:** Dementia, Alzheimer’s disease, Diagnosis, Prevalence, Incidence, Epidemiology, Primary care, Hospital, Long-term care, Prescription drug

## Abstract

**Background:**

Determining the epidemiology of dementia among the population as a whole in specific jurisdictions – including the long-term care population–is essential to providing appropriate care. The objectives of this study were to use linked administrative databases in the province of Saskatchewan to determine the 12-month incidence and prevalence of dementia for the 2012/13 period (1) among individuals aged 45 and older in the province of Saskatchewan, (2) according to age group and sex, and (3) according to diagnosis code and other case definition criteria.

**Methods:**

We used a population-based retrospective cohort study design and extracted data from 10 provincial health databases linked by a unique health services number. The cohort included individuals 45 years and older at first identification of dementia between April 1, 2001 and March 31, 2013 based on case definitions met within any one of four administrative health databases (Hospital Discharge Abstracts, Physician Service Claims, Prescription Drug, and RAI-MDS, i.e., Long-term Care).

**Results:**

A total of 3,270 incident cases of dementia (7.28 per 1,000 PAR) and 13,012 prevalent cases (28.16 per 1,000 PAR) were identified during 2012/13. This study found the incidence rate increased by 2.8 to 5.1 times and the prevalence rate increased by 2.6 to 4.6 times every 10 years after 45 years of age. Overall, the age-standardised incidence rate was significantly lower among females than males (7.04 vs. 7.65 per 1,000 PAR) and the age-standardised prevalence rate was significantly higher among females than males (28.92 vs. 26.53 per 1,000 PAR). Over one-quarter (28 %) of all incident cases were admitted to long-term care before a diagnosis was formally recorded in physician or hospital data, and nearly two-thirds of these cases were identified at admission with impairment at the moderate to very severe level or a disease category of Alzheimer’s disease/other dementia.

**Conclusions:**

Linking multiple sources of registry data contributes to our understanding of the epidemiology of dementia across multiple segments of the population, inclusive of individuals residing in long-term care. This information is foundational for public awareness and policy recommendations, health promotion and prevention strategies, appropriate health resource planning, and research priorities.

**Electronic supplementary material:**

The online version of this article (doi:10.1186/s12877-015-0075-3) contains supplementary material, which is available to authorized users.

## Background

Worldwide, it is estimated that there is one incident case of dementia every 4 seconds, or 7.7 million incident cases each year [1]. The most recent estimates show that 44 million people now live with dementia, which is projected to increase to 135 million by 2050 [2]. Historically low fertility rates and improved life expectancy are expected to be the main drivers of population aging [3], with the absolute number of older adults aged 65 years and older tripling worldwide from 524 million in 2010 to 1.5 billion in 2050 [4]. Among individuals aged 60 years and over in 2010, dementia prevalence was between 5-7 % worldwide; dementia onset before the age of 65 (i.e., early onset dementia) was estimated to account for 6-9 % of all prevalent cases [2]. International recognition of the association between population aging and the growing numbers of individuals with chronic and degenerative diseases has resulted in greater attention on dementia as a significant global health challenge [2].

As a result of the first-ever G8 dementia summit held in 2013, a new Global Envoy for Dementia Innovation was appointed and an agreement was reached to collaborate internationally on dementia research that included an investment in ‘making timely diagnosis and early intervention feasible, affordable and cost effective’ as one of five priorities [5]. Many national dementia plans propose initiatives to improve detection and formal diagnosis. The more prominent of these initiatives involve building capacity among health care professionals to diagnose and treat dementia in its early stages, improving public awareness of symptoms and stigma associated with resistance to help-seeking, and improving the availability and accessibility of diagnostic and post-diagnosis resources [6–8].

Three separate systematic reviews have found that between 31 % and 69 % of patients with dementia in primary care do not receive a formal, i.e., documented diagnosis; formal diagnosis is less likely to occur among mild than moderate or severe cases [9–11]. Sternberg and colleagues [12] found that two of every three community-dwelling Canadian seniors (64 %) with dementia were undetected, defined as “never having seen a physician for memory problems”. Key factors that have been identified as possible barriers to formal diagnosis include patient and caregiver issues such as lack of symptom awareness, lack of support to seek help, and attitudes such as resistance to receiving a diagnosis and perceived stigma; health care professional attitudes such as poor knowledge and adherence to clinical practice guidelines, diagnostic uncertainty and difficulties in diagnosis disclosure, and skepticism regarding the effectiveness of treatment (i.e., therapeutic nihilism [13]); and health system factors such as limited time to conduct consultations and poor access to diagnostic information and resources [10, 11, 14].

An essential step in providing appropriate care for people with dementia is ascertaining the burden of the disease among the population as a whole – including the long-term care population – in specific jurisdictions. Findings from field studies (i.e., two-phase studies with screening followed by a structured clinical evaluation) contribute to current evidence in this regard. However, field studies of dementia epidemiology typically do not combine community-dwelling and institutionalized populations e.g., [15–18], including one of the most comprehensive reports of global dementia epidemiology [1]. Some field studies have combined these populations e.g., [19, 20]. Registry studies (i.e., based on administrative health data), such as the current study, are useful for several reasons, foremost being the information upon which to base policy recommendations, public awareness activities, health promotion and prevention strategies, health resource planning, and research priorities. The current study included a lower age cut-off of 45 years in order to identify the incidence and prevalence of ‘early onset dementia’, that is dementia in individuals younger than age 65 years. This information is useful for planning the health and social care services necessary to address the unique needs of individuals with early onset dementia and their families [21]. In addition to providing critical policy information, the current study includes a comparison of specific diagnosis codes and other criteria used in our case definition with other similar recent studies, and as such offers: (1) increased awareness of the nature and availability of codes to record disease diagnoses; (2) improved understanding of the role of diagnosis codes in estimating disease incidence and prevalence; (3) opportunity for transparency in the development of definitions to identify cases and potential for common agreement among researchers on such definitions; and (4) comparisons of rates and risk factors among regions with access to similar data [22, 23]. The objectives of the present study were to use linked administrative databases in the province of Saskatchewan to determine the 12-month incidence and prevalence of dementia (1) among individuals aged 45 and older in the province of Saskatchewan, (2) according to age group and sex, and (3) according to diagnosis code and other case definition criteria.

## Methods

### Setting

The province of Saskatchewan (Canada) covers 651,000 km^2^ and has a population of over 1.08 million [24]. In 2012, two cities in the province each had populations over 100,000 (census metropolitan areas [CMAs]) that together accounted for 46.6 % of the population [25]; communities and areas outside of these CMAs contributed 53.4 % of the population. Individuals aged 0 to 14 years accounted for 18.8 % (204,436), those 15 to 44 years of age made up 40.4 % (439,041), individuals 45 to 64 years of age accounted for 26.4 % (287,286), and those aged 65 years and older made up 14.4 % (156,873) of the population [24]. The median age of the provincial population was 37.1 years in 2012 [26].

In Canada’s publicly funded system of universal health care, each of Canada’s 10 provinces and 3 territories is responsible for the provision of ‘medically necessary hospital and physician services’ to its citizens [27]. Determination of the services that are medically necessary, i.e., fully covered services, is the separate responsibility of each province and territory. In Saskatchewan, many services beyond inpatient and outpatient care provided by physicians are fully covered, including immunizations for children, physiotherapy in hospitals and special care homes, and mental health services [28]. All residents of Saskatchewan are eligible for health insurance to receive fully covered services with the exception of those covered by federal health insurance (e.g., federal prison inmates, members of the Canadian Forces and Royal Canadian Mounted Police) and individuals who do not meet the residency requirements of Saskatchewan (i.e., those who have lived in the province for a period of less than three months or have moved elsewhere for a period of more than three months) [29]. In addition, the Prescription Drug Plan excludes the Registered Indian population and other residents whose costs are covered by another government body [30]. Saskatchewan residents who receive health insurance benefits comprise the ‘covered population’.

### Data sources

This analysis used data extracted from 10 provincial health databases linked by a unique personal health services number assigned to individuals eligible for health insurance benefits [30]. Home Care data were not available for analysis. The databases were accessed, linked, and analysed by researchers at the Saskatchewan Health Quality Council (HQC) through a formal data sharing agreement between HQC and the Saskatchewan Ministry of Health.

The *Hospital Discharge Abstract Database* includes patient information, most responsible diagnosis for hospitalization, other diagnoses, principal procedure, other procedures, accident code, and hospital discharge dates. Prior to April 1, 2002, four-digit ICD-9 codes were used to record a maximum of 16 diagnoses per record. Five-digit ICD-10-CA codes were introduced April 1, 2001, after which time approximately 30 % of hospitals in Saskatchewan continued to use ICD-9 codes. By April 1, 2002, the transition to ICD-10-CA codes was complete and all hospitals were using this 5-digit coding system to record up to 25 diagnoses per record.

The *Physician Services Claims Database* includes information used by physicians to claim payment from the provincial government for services provided to patients. Patient information is included, as well as service information such as date, fee code, type, diagnosis code associated with service (maximum of one diagnosis code per service claim), location, and payment information [30]. Physicians who are remunerated on a non-fee-for-service basis are also expected to submit similar ‘shadow’ or ‘dummy’ billing claims. Although approximately 16 % of full-time equivalent physicians receive payment on a non-fee-for-service basis, the completeness of the shadow billing claims is unknown [31]. The *Physician Mobility File* contains physicians’ identification numbers and specialties. More than 70 specialty categories based on physician certification are available [31].

The two Prescription Drug Databases (*ALLDIN* and *Historical Claims*) include information regarding the dispensing pharmacy as well as information about the drug dispensed such as classification of drug, drug identification number (DIN), type and class, generic and brand names, strength and dosage, date and quantity dispensed, and cost. Only those drugs listed in the Saskatchewan Formulary are eligible for coverage under the Saskatchewan Drug Plan. The prescription costs for the Registered Indian population are paid by the federal government and therefore prescriptions for these individuals are not included in the Prescription Drug Database [30]. The Registered Indian population accounted for approximately 13 % of the Saskatchewan population in 2012 [32].

The *Resident Assessment Instrument – Minimum Data Set* (*RAI-MDS*), (i.e., Long-term Care Database), contains information gathered from the assessment of individuals at the time of admission to a residential care facility and at regular three-month intervals [33]. Residents also receive an assessment if their clinical status changes significantly. Included in the data are: residents’ identification and background information, disease diagnoses, health conditions, skin condition, medication list, as well as measures of mood and behaviour, vision, cognition, communication and hearing, accidents, physical functioning, clinical management, continence, oral and nutrition status, activity patterns, and psychosocial well-being. Admission and quarterly assessment data were included in the present study. These data have been used extensively for research and their validity for use in research has been confirmed in multiple studies [34–36].

The *Institutional Supportive Care Data Set* contains information on the facilities that house long-term residents. The *Person Health Registration System*, *Saskatchewan Resident Geography*, and *Vital Statistics* databases contain information regarding status and dates of insurance coverage, gender, dates of birth and death, urban vs. rural residence, and health region of residence.

### Cohort

#### Selection of case definition criteria

A three-stage process was used to develop the case definition algorithm. The first stage consisted of conducting an overview of recent Canadian studies that employed administrative data to determine the dementia incidence or prevalence in the general population, to identify previous criteria used to define cases of dementia (see Table 1 and Additional file 1). Canadian studies were chosen for comparison due to the similarity of features among administrative databases across the country, specifically the availability of physician services databases in 11 of 13 provinces, the similar duration of data availability, and the use of similar diagnosis and procedure codes across provinces [31]. The second stage involved separate reviews of the case definition criteria employed in the selected Canadian studies by: (1) the Rural and Remote Memory Clinic team consisting of the director (DM), neurologist (AK) and neuropsychologists (MC and MO), and (2) a Steering Committee that included family physicians, nurse practitioners, as well as leadership from the Alzheimer Society of Saskatchewan and health regions. The final stage consisted of consolidating the reviews and reaching consensus among the clinical team members on the diagnosis codes and other criteria that comprised the case definition algorithm.

**Table 1:** Case definition algorithms employed to identify dementia in administrative health databases, by Canadian study

Study/Institute	Case definition algorithm^b^	Age group	Timeframe
Present study^a^	≥1 physician visits or ≥ 1 hospitalizations or ≥ 1 prescriptions for a cholinesterase inhibitor or [a RAI-MDS CPS score of ≥ 2 and/or (a disease category of Alzheimer’s disease or dementia other than Alzheimer’s disease)]	≥45 years	1 year (2012–2013)
Manitoba Centre for Health Policy [38]^c^	≥1 physician visits or ≥ 1 hospitalizations	≥55 years	Not applicable^b^
Chartier et al. [39]	≥1 physician visits or ≥ 1 hospitalizations	≥55 years	5 years (2004–2009)
Martens et al. [40]	≥1 physician visits or ≥ 1 hospitalizations	≥55 years	5 years (2002–2007)
Fransoo et al. [41]	≥1 physician visits or ≥ 1 hospitalizations	≥55 years	5 years (1996–2001) and 5 years (2001–2006)
Gill et al. [42]	≥1 physician visits or ≥ 1 hospitalizations or any cholinesterase inhibitor prescription	66-105 years	5 years (2002–2007) for physician and hospital data; 1 year for prescription data (2006–2007)
Jacklin et al. [43]	≥1 physician visits	all ages	1 year (2008–2009)
Jacklin and Walker [44]	≥2 physician visits or ≥ 1 hospitalizations	≥60 years	1 year (2006–2007)

^a^See ‘Case definition criteria’ for the complete case definition algorithm employed in the present study

^b^See Additional file 1 for the diagnosis codes and other criteria employed to identify dementia cases in the studies

^c^Research institute

#### Case definition criteria

**Fig. 1:**
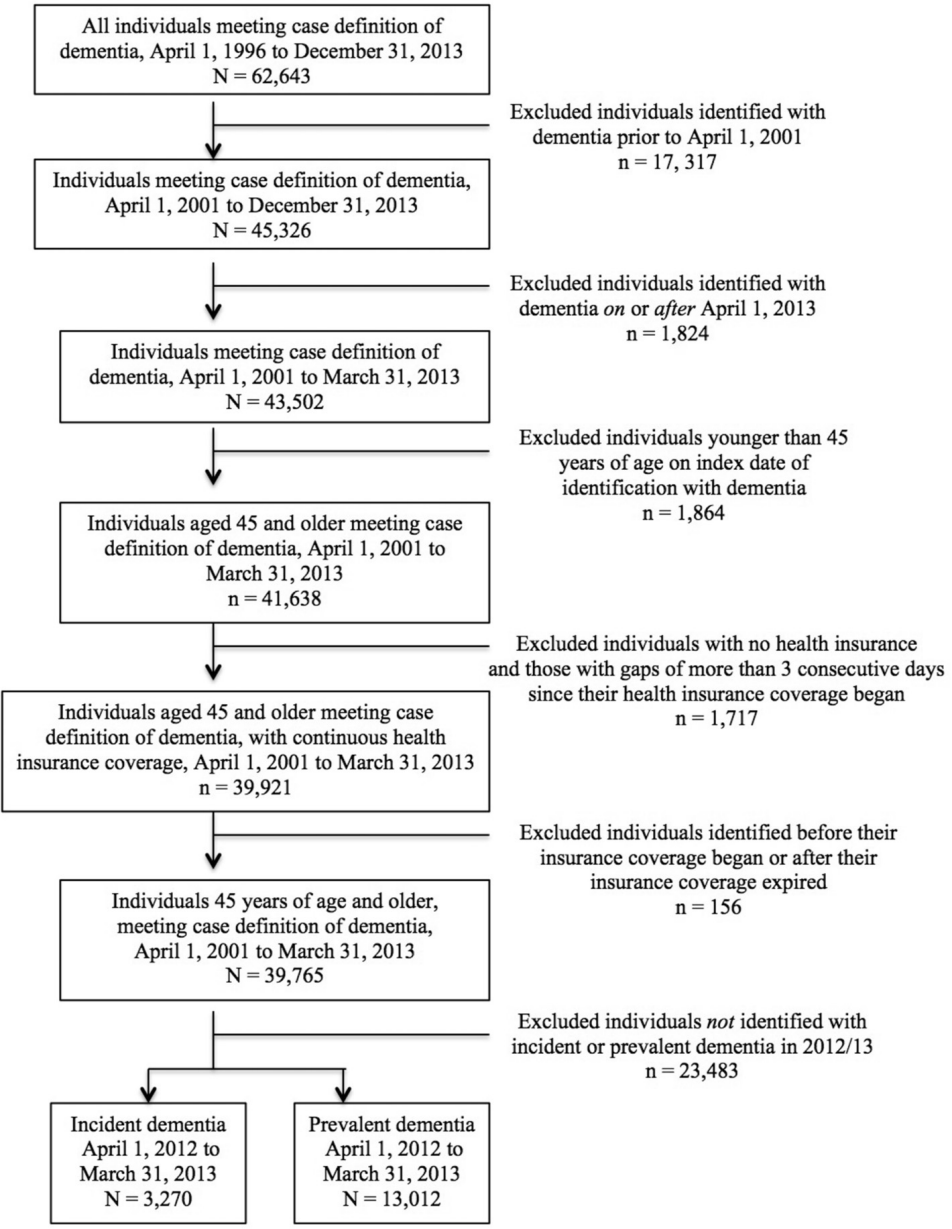
Identification of incident and prevalent cases of dementia (2012/13) based on case definition criteria

The cohort included individuals aged 45 years and older at their first-ever recorded identification of dementia (i.e., index date) between April 1, 2001 and March 31, 2013 in one of four administrative health databases (Hospital Discharge Abstracts, Physician Service Claims, Prescription Drug, and RAI-MDS, i.e., Long-term Care). Eligible individuals had continuous health insurance coverage from the start of their insurance until one of the following: March 31, 2013, the expiration of their insurance, or their death. Individuals with gaps of no more than 3 consecutive days in their coverage were considered to have continuous health insurance. The case definition algorithm employed in the present study is provided in Table 1 and the identification of the study cohort is shown in Fig. 1. The specific criteria applied to the four administrative health databases were as follows:

1. Hospital Discharge Abstracts (ICD-10-CA codes: F00, F01, F02, F03, F04, F05.1, F06.8, F06.9, F09, F10.6, F10.7, F18.6, F18.7, F19.6, F19.7, G30, G31.0, G31.1, G91, R54);
2. Physician Services Claims (ICD-9 codes: 290, 294, 331, 797);
3. Prescription Drug (Aricept DINs: 02232043, 02232044; Exelon DINs: 02242115–02242118, 02245240; Reminyl DINs: 02244298–02244300, 02266717, 02266725, 02266733);
4. Long-term Care, i.e., RAI-MDS (Cognitive Performance Scale (CPS) score of 2 and over, indicating mild to very severe impairment) [37] and/or a disease category of Alzheimer’s disease *or* dementia other than Alzheimer’s disease.

If individuals had the same index date in two or more databases, they were identified first in the physician database, then in the hospital, then RAI-MDS, and lastly the drug database. If individuals had a CPS score of 2 or over recorded on the same index date as a disease category of Alzheimer’s disease and/or dementia other than Alzheimer’s disease, they were identified first by the CPS score, then by the disease category of Alzheimer’s disease, and last by the disease category of dementia other than Alzheimer’s disease.

As shown in Table 1, all of the previous studies that employed administrative data to define dementia used physician data, and most often, also used hospital data [38–44]. The case definitions used in the majority of these studies required a minimum of one physician visit or one hospitalization. Also, the lower age cut-offs varied from no cut-off (i.e., all ages included) to 66 years.

Diagnosis codes and other criteria employed in the case definition algorithm for the present study were selected with a goal to achieve sensitivity over specificity, given substantial evidence that dementia underdiagnosis is a significant and global phenomenon [7, 9, 45]. Certain neurologic conditions, such as Alzheimer’s disease and Parkinson’s disease, do not have a diagnostic test for confirmation purposes and it may be more challenging for physicians to assign a diagnosis code to these conditions compared to other neurologic conditions [46]. A recent systematic review of studies that validated the use of specific ICD-9 and ICD-10 codes in administrative health data found eight studies that tested a total of 21 case definitions of dementia against a reference standard [46]. On average, specificity was higher than 84 % (range of 56.3 % to 100 %), while sensitivity ranged from 8 % to 86.5 %. While case definitions of dementia that employ numerous ICD-9 and ICD-10 diagnosis codes may be highly sensitive to identifying people who actually have dementia, the challenge is that individuals without dementia may also be captured in these same case definitions. In the present study, sensitivity was pursued by employing a case definition algorithm that targeted individuals who may not have had a formal diagnosis in physician or hospital data, but who may have sufficiently satisfied other criteria to be included in the dementia cohort. These other criteria related to prescription drug and long-term care services, and are described in the case definition algorithm (Table 1 and Additional file 1).

##### Hospital data

As shown in Additional file 1, the ICD-9 codes used to identify dementia cases in hospital data during the ‘washout period’ of 1996 to 2001 (i.e., run-in period to ensure that cases identified after April 1, 2001 were in fact incident cases, and had not been previously identified) in the present study were identical to codes recommended by the Manitoba Centre for Health Policy (MCHP) [38], and used by Chartier et al. [39]. Recent recommendations from MCHP exclude most diagnosis codes that refer to the involvement of alcohol or drugs. Therefore, the present study excluded most ICD-9 codes with elements of alcohol or drugs that did not also contain specific reference to a dementia (i.e., 291.3, 291.4, 291.5, 291.8, 291.9, 292.0, 292.1, 292.2, 292.9) in contrast to earlier studies [40, 41].

Following MCHP recommendations, and in accordance with earlier studies [40, 41], the present study included ICD-10-CA codes of mental and behavioural disorders due to: ‘alcohol, residual and late-onset psychotic disorder’ (F10.7), ‘volatile solvents, residual and late-onset psychotic disorder’ (F18.7), and ‘multiple drug use and use of other psychoactive substances, residual and late-onset psychotic disorder’ (F19.7). In contrast to MCHP recommendations but still in accordance with earlier studies [40, 41], the present study included hospital ICD-10-CA codes (2001–2013) of mental and behavioural disorders due to: ‘alcohol, amnesic syndrome’ (F10.6), ‘volatile solvents, amnesic syndrome’ (F18.6), and ‘multiple drug use and use of other psychoactive substances, amnesic syndrome’ (F19.6).

##### Physician data

The present study followed MCHP recommendations regarding the inclusion of ICD-9 codes in physician data (2001–2013). Diagnoses of ‘alcohol psychoses’ (291) and ‘drug psychoses’ (292) used in earlier studies [40, 41] were not used in the present study. The Saskatchewan physician data contain 3-digit ICD-9 codes, and specific dementia-related diagnoses that are specified in the 4^th^ digit of ICD-9 that we would otherwise have included could not be included in the case definition (i.e., 291.1 ‘Korsakov’s psychosis’ and 291.2 ‘other alcoholic dementia’).

##### Prescription drug data

Three cholinesterase inhibitors, Aricept, Exelon, and Reminyl, were included in the case definition algorithm for the present study. Aricept (donepezil), Reminyl (galantamine), and Exelon (rivastigmine) are the only three cholinesterase inhibitors currently prescribed in Canada [47]. Cholinesterase inhibitors are considered first-line pharmacotherapy for the purpose of improving symptoms associated with dementia due to Alzheimer’s disease [AD] [48]. While other medications may be used to treat AD (e.g., memantine) [47], these three cholinesterase inhibitors are the most common medications used and are typically prescribed only for the treatment of dementia.

##### Long-term care data

Individuals whose index date was within 30 days after their date of admission to long-term care were considered to be identified ‘at admission’ to account for the reality that some staff, particularly in smaller facilities, were not able to conduct a resident’s assessment on the same day as admission. With respect to the use of RAI-MDS (long-term care) data in the case definition algorithm, a Cognitive Performance Scale score of 2 or higher was required, and/or a disease category of Alzheimer’s disease or dementia other than Alzheimer’s disease. The disease categories are based on transfer documentation, medical records, and information provided by the patient that has been verified by a physician [33]. A CPS score of 2 or higher is equivalent to an average Mini Mental State Examination score of 19 or lower [49], indicating possible mild to very severe impairment [37] and dementia at the moderate to severe stage [50]. Validated against physician diagnosed dementia in a sample of older hospitalized patients [51], a CPS score of 2 or higher had moderate sensitivity (0.68) and very good specificity (0.92). Validated against the Cambridge Examination for Mental Disorders of the Elderly-Revised (CAMDEX-R) in a sample of older nursing home residents, a CPS score of 2 or higher had good sensitivity (0.81) and specificity (0.80) for the detection of cognitive impairment [52].

### Measures

Three independent variables were included in the analysis: sex, age, and administrative health database. The age groupings were 45–54, 55–64, 65–74, 75–84, and 85 years and over. Hospital, physician, prescription drug, and long-term care represented the four administrative health databases.

### Statistical analysis

All analyses were conducted with SAS 9.3 (SAS Institute Inc, Cary NC). The number of incident dementia cases was calculated for each 12-month period from April 1, 2001 to March 31, 2013. Only the 12-month period of 2012–2013 is reported in this paper. New cases of dementia included individuals who met the case definition criteria and did not have a previous identification of dementia during the ‘washout’ period between April 1, 1996 and March 31, 2001 in any of the following databases: (1) Hospital Discharge Abstracts (ICD-9 codes: 290, 291.1, 291.2, 292.8, 294, 331, and 797); (2) Physician Services Claims Database (ICD-9 codes: 290, 294, 331, 797); and (3) Prescription Drug (Aricept, Exelon, or Reminyl).

The numerator for the 2012/2013 incidence rate per 1,000 population at risk (PAR) was the number of people who were alive on April 1, 2012 and who met the case definition of dementia between April 1, 2012 and March 31, 2013. The denominator consisted of the population at risk of incident dementia, which included all individuals in the covered population aged 45 years or older on April 1, 2012 with at least one day of health insurance coverage for the 12-month period, after removing individuals with prevalent dementia for the same period.

Dementia prevalence was calculated for each 12-month period from 2002 to 2013, April 1 to March 31 inclusive. Prevalent cases of dementia included individuals who met the case definition criteria for each 12-month period. Only data for the 2012–2013 12-month period are presented in this paper. The numerator for the 2012/2013 prevalence rate per 1,000 PAR was the number of individuals who met the case definition criteria at any time prior to April 1, 2012 and were alive on April 1, 2012. The denominator was the number of individuals at risk for prevalent dementia, that is, all individuals in the covered population aged 45 years or older on April 1, 2012 with at least one day of health insurance coverage for the 12-month period.

Sex-specific incidence and prevalence rates were adjusted for age distribution using the age structure of the total cohort. Female to male age-standardised incidence and prevalence rate ratios (RR) were calculated by dividing female rates by male rates, and 95 % confidence intervals (CI) were calculated for all crude rates, age-standardised rates, and rate ratios.

### Ethics

This study received ethics approval from the University of Saskatchewan Biomedical Research Ethics Board (Bio-REB #12-339).

## Results

### Incidence by sex and age group

Overall, 3,270 incident cases of dementia were identified among adults aged 45 years and older in Saskatchewan for the 2012/13 period (Fig. 1). The majority of these individuals (98.2 %) had continuous health insurance coverage for five years prior to identification of dementia. As shown in Table 2, the 12-month incidence rate among all adults was 7.28 per 1,000 population at risk (PAR). Adults under 65 years contributed 7.55 % of the incident cases (247/3,270), those aged 65 to 84 contributed 41.07 % (1,343/3,270), and 51.38 % (1,680/3,270) were contributed by adults aged 85 and older. The sharpest escalation in the incidence rate occurred at age 75, increasing by 5.07 times (20.03 per 1,000 PAR) compared to the group aged 65 to 74 (3.95 per 1,000 PAR). Among all adults, the incidence rate increased 152 times between the groups aged 45 to 54 and 85 years and older (0.46 vs. 69.73 per 1,000 PAR).

**Table 2:** 12-month incidence of dementia among adults 45 years of age and older, Saskatchewan, by age group (April 1, 2012 to March 31, 2013)

Age group	n	PAR	Rate per 1,000 PAR (95 % CI)
45-54	70	153,189	0.46 (0.35-0.56)
55-64	177	137,916	1.28 (1.07-1.47)
65-74	329	83,198	3.95 (3.53-4.38)
75-84	1,014	50,616	20.03 (18.80-21.27)
85+	1,680	24,093	69.73 (66.40-73.06)
Total	3,270	449,012	7.28 (7.03-7.53)

As shown in Table 3, a total of 1,887 incident cases in females and 1,383 incident cases in males were observed in adults aged 45 years and older. The number of female and male incident cases under 75 years was similar (287 vs. 289); after age 75, female incident cases outnumbered male cases (1,600 vs. 1,094). After adjustment for age, the female to male standardised incidence rate ratio was significantly different in the 85 and older age group only (RR: 0.90, CI: 0.817-0.997, p < 0.05) (Table 4). Among all age groups combined, the standardised incidence rate was 8 % lower among females than males (7.04 vs. 7.65 per 1,000 PAR); the female to male rate ratio of 0.92 (95 % CI: 0.859-0.986) was significant (p < 0.05).

**Table 3:** 12-month incidence of dementia among adults 45 years of age and older, Saskatchewan, by gender and age group (April 1, 2012 to March 31, 2013)

Age group	Female				Male			
	n	PAR	Crude rate per 1,000 PAR (95 % CI)	Age-standardised rate per 1,000 PAR (95 % CI)	n	PAR	Crude rate per 1,000 PAR (95 % CI)	Age-standardised rate per 1,000 PAR (95 % CI)
45-54	37	75,597	0.49 (0.33-0.65)	0.49 (0.33-0.65)	33	77,592	0.43 (0.28-0.57)	0.43 (0.28-0.57)
55-64	85	67,958	1.25 (0.98-1.52)	1.25 (0.99-1.52)	92	69,958	1.32 (1.05-1.58)	1.32 (1.05-1.58)
65-74	165	42,193	3.91 (3.31-4.51)	3.88 (3.29-4.47)	164	41,005	4.00 (3.39-4.61)	4.02 (3.41-4.64)
75-84	539	27,767	19.41 (17.77-21.05)	19.26 (17.63-20.88)	475	22,849	20.79 (18.92-22.66)	21.05 (19.16-22.94)
85+	1,061	15,267	69.50 (65.31-73.68)	67.13 (63.09-71.17)	619	8,826	70.13 (64.61-75.66)	74.38 (68.52-80.24)
Total	1,887	228,782	8.25 (7.88-8.62)	7.04 (6.72-7.36)	1,383	220,230	6.28 (5.95-6.61)	7.65 (7.25-8.06)

**Table 4:** 12-month age-standardised incidence rates and female/male rate ratios among adults 45 years of age and older, Saskatchewan, by gender and age group (April 1, 2012 to March 31, 2013)

Age group	Female age-standardised rate per 1,000 PAR	Male age-standardised rate per 1,000 PAR	Rate ratio	SD (RR)	95 % CI
45-54	0.49	0.43	1.14	0.24	0.712-1.822
55-64	1.25	1.32	0.95	0.15	0.705-1.272
65-74	3.88	4.02	0.97	0.11	0.778-1.198
75-84	19.26	21.05	0.91	0.06	0.809-1.035
85+	67.13	74.38	0.90	0.05	0.817-0.997
Total	7.04	7.65	0.92	0.04	0.859-0.986

### Prevalence by sex and age group

A total of 13,012 prevalent dementia cases among adults aged 45 years and older in Saskatchewan were identified by administrative data analysis for the 2012/13 period (Fig. 1). Adults under 65 years contributed 8.35 % of prevalent cases (1,087/13,012), those aged 65 to 84 accounted for 39.03 % (5,078/13,012), and adults aged 85 years and older accounted for 52.62 % (6,847/13,012). As shown in Table 5, the prevalence rate increased steadily with age, with the largest increase at 4.57 times occurring between the age groups 45 to 54 and 55 to 64 (1.38 vs. 6.31 per 1,000 PAR). Overall, the prevalence rate was 160 times higher among adults aged 85 and older than among those 45 to 54 years of age (221.30 vs. 1.38 per 1,000 PAR).

**Table 5:** 12-month prevalence of dementia among adults 45 years of age and older, Saskatchewan, by age group (April 1, 2012 to March 31, 2013)

Age group	n	PAR	Rate per 1,000 PAR (95 % CI)
45-54	211	153,400	1.38 (1.19-1.56)
55-64	876	138,792	6.31 (5.89-6.73)
65-74	1,391	84,589	16.44 (15.58-17.31)
75-84	3,687	54,303	67.90 (65.71-70.09)
85+	6,847	30,940	221.30 (216.06-226.54)
Total	13,012	462,024	28.16 (27.68-28.65)

There were a similar number of female and male prevalent cases under age 75 (1,250 vs. 1,228), but a greater number of female than male prevalent cases after age 75 (6,849 vs. 3,685) (Table 6). Among adults aged 45 years and older, a total of 8,099 prevalent cases in females and 4,913 prevalent cases in males were observed. After adjusting for age, Table 7 shows that the only age group for which the female to male standardised prevalence rate ratio was significant was 85 and older (RR: 1.18, 95 % CI: 1.123-1.245, p < 0.05). Among all age groups combined, the standardised prevalence rate was 9 % higher among females than males (28.92 vs. 26.53 per 1,000 PAR); the female to male prevalence rate ratio was significant at 1.09 (95 % CI: 1.052-1.129, p < 0.05)

**Table 6:** 12-month prevalence of dementia among adults 45 years of age and older, Saskatchewan, by gender and age group (April 1, 2012 to March 31, 2013)

Age group	Female				Male			
	n	PAR	Crude rate per 1,000 PAR (95 % CI)	Age-standardised rate per 1,000 PAR (95 % CI)	n	PAR	Crude rate per 1,000 PAR (95 % CI)	Age-standardised rate per 1,000 PAR (95 % CI)
45-54	110	75,707	1.45 (1.18-1.72)	1.45 (1.18-1.72)	101	77,693	1.30 (1.05-1.55)	1.30 (1.05-1.56)
55-64	446	68,404	6.52 (5.91-7.13)	6.52 (5.91-7.12)	430	70,388	6.11 (5.53-6.69)	6.11 (5.53-6.69)
65-74	694	42,887	16.18 (14.98-17.39)	16.12 (14.92-17.32)	697	41,702	16.71 (15.47-17.95)	16.79 (15.54-18.03)
75-84	2,034	29,801	68.25 (65.29-71.22)	67.56 (64.62-70.50)	1,653	24,502	67.46 (64.21-70.72)	68.24 (64.95-71.53)
85+	4,815	20,082	239.77 (232.99-246.54)	231.94 (225.39-238.49)	2,032	10,858	187.14 (179.01-195.28)	196.16 (187.63-204.69)
Total	8,099	236,881	34.19 (33.45-34.93)	28.92 (28.29-29.55)	4,913	225,143	21.82 (21.21-22.43)	26.53 (25.78-27.27)

**Table 7:** 12-month age-standardised prevalence rates and female/male rate ratios among adults 45 years of age and older, Saskatchewan, by gender and age group (April 1, 2012 to March 31, 2013)

Age group	Female age-standardised rate per 1,000 PAR	Male age-standardised rate per 1,000 PAR	Rate ratio	SD (RR)	95 % CI
45-54	1.45	1.30	1.12	0.138	0.851-1.461
55-64	6.52	6.11	1.07	0.068	0.935-1.218
65-74	16.12	16.79	0.96	0.054	0.864-1.067
75-84	67.56	68.24	0.99	0.033	0.928-1.056
85+	231.94	196.16	1.18	0.027	1.123-1.245
Total	28.92	26.53	1.09	0.018	1.052-1.129

The 12-month prevalence rate was 3.9 times the 12-month incidence rate (28.16 vs. 7.28 per 1,000 PAR), indicating an average duration of survival from time of identification by diagnosis or other criteria of 3.9 years. Upon further examination, females survived an average of 4.11 years from time of identification (28.92/7.04 per 1,000 PAR) and males survived an average of 3.47 years (26.53/7.65 per 1,000 PAR).

### Incidence and prevalence by diagnosis code and other criteria

As shown in Table 8, the greatest proportion of all 12-month incident cases in 2012/13 were first identified in long-term care (34.98 %) followed closely by a diagnosis in physician services claims (29.94 %), and a diagnosis in hospital (28.53 %). Of note, 6.54 % of all incident cases were first identified as a result of a cholinesterase inhibitor prescription. Five separate diagnosis codes and other criteria represented three-quarters of all incident cases (73.59 %): an ICD-10-CA diagnosis of ‘unspecified dementia’ in hospital (18.69 %), an ICD-9 diagnosis of ‘other cerebral degenerations’ in physician services claims (14.40 %), CPS Scale scores indicating mild (13.52 %) or moderate (13.49 %) impairment in long-term care, and an ICD-9 diagnosis of ‘senile and presenile organic psychotic conditions’ (13.49 %) in physician services claims.

**Table 8:** Incidence and prevalence of dementia among adults 45 years of age and older, by diagnosis code and other criteria at first identification, Saskatchewan (April 1, 2012 to March 31, 2013)

Database and diagnosis code/other criteria	Description of diagnosis code/other criteria	Incident cases			Prevalent cases		
		n	Rate per 100 with incident dementia^a^	Rate per 10,000 PAR^b^	n	Rate per 100 with prevalent dementia^c^	Rate per 10,000 PAR^d^
Hospital Discharge Abstract Database (ICD-10-CA Codes)							
F00.0	Dementia in Alzheimer’s Disease with early onset	<6	*	*	9	0.07	0.19
F00.1	Dementia in Alzheimer’s Disease with late onset	<6	*	*	16	0.12	0.35
F00.2	Dementia in Alzheimer’s Disease, atypical or mixed type	<6	*	*	19	0.15	0.41
F00.9	Dementia in Alzheimer’s Disease, unspecified	38	1.16	0.85	156	1.20	3.38
F01.0	Vascular dementia of acute onset	<6	*	*	<6	*	*
F01.1	Multifarct dementia	10	0.31	0.22	26	0.20	0.56
F01.2	Subcortical vascular dementia	<6	*	*	7	0.05	0.15
F01.3	Mixed cortical and subcortical vascular dementia	<6	*	*	<6	*	*
F01.8	Other vascular dementia	<6	*	*	14	0.11	0.30
F01.9	Vascular dementia, unspecified	47	1.44	1.05	189	1.45	4.09
F02.0	Dementia in Pick’s disease	<6	*	*	14	0.11	0.30
F02.1	Dementia in Creutzfeldt-Jakob disease	<6	*	*	0		
F02.2	Dementia in Huntington’s disease	0			<6	*	*
F02.3	Dementia in Parkinson’s disease	22	0.67	0.49	68	0.52	1.47
F02.4	Dementia in human immunodeficiency virus HIV disease	0			<6	*	*
F02.8	Dementia in other specified diseases classified elsewhere	<6	*	*	16	0.12	0.35
F03	Unspecified dementia	611	18.69	13.61	1,737	13.35	37.60
F04	Organic amnesic syndrome, not induced by alcohol and other psychoactive substances	0			<6	*	*
F05.1	Delirium superimposed on dementia	54	1.65	1.20	158	1.21	3.42
F06.8	Other specified mental disorders due to brain damage and dysfunction and to physical disease	0			15	0.12	0.32
F06.9	Unspecified mental disorder due to brain damage and dysfunction and to physical disease	<6	*	*	108	0.83	2.34
F09	Unspecified organic or symptomatic mental disorder	<6	*	*	14	0.11	0.30
F10.6	Mental and behavioural disorders due to use of alcohol, amnesic syndrome	13	0.40	0.29	61	0.47	1.32
F10.7	Mental and behavioural disorders due to use of alcohol, residual and late-onset psychotic disorder	8	0.24	0.18	59	0.45	1.28
F18.6	Mental and behavioural disorders due to use of volatile solvents, amnesic syndrome	0			0	0.00	0.00
F18.7	Mental and behavioural disorders due to use of volatile solvents, residual and late-onset psychotic disorder	0			0	0.00	0.00
F19.6	Mental and behavioural disorders due to multiple drug use and use of other psychoactive substances, amnesic syndrome	0			<6	*	*
F19.7	Mental and behavioural disorders due to multiple drug use and use of psychoactive substances, residual and late-onset psychotic disorder	0			<6	*	*
G30.0	Alzheimer’s disease with early onset	0			6	0.05	0.13
G30.1	Alzheimer’s disease with late onset	<6	*	*	10	0.08	0.22
G30.8	Other Alzheimer’s disease	0			18	0.14	0.39
G30.9	Alzheimer’s disease, unspecified	24	0.73	0.53	141	1.08	3.05
G31.0	Circumscribed brain atrophy	0			<6	*	*
G31.1	Senile degeneration of brain, not elsewhere classified	<6	*	*	7	0.05	0.15
G91.0	Communicating hydrocephalus	<6	*	*	<6	*	*
G91.2	Normal-pressure hydrocephalus	8	0.24	0.18	49	0.38	1.06
R54	Senility	74	2.26	1.65	161	1.24	3.48
Total Hospital Discharge Abstract Database (ICD-10-CA Codes)		933	28.53	20.78	3,102	23.84	67.14
Physician Services Claims Database & Physician Characteristics Database (ICD-9 Codes)							
290	Senile and presenile organic psychotic conditions	441	13.49	9.82	1,709	13.13	36.99
294	Other organic psychotic conditions chronic	23	0.70	0.51	107	0.82	2.32
331	Other cerebral degenerations	471	14.40	10.49	3,205	24.63	69.37
797	Senility without mention of psychosis	44	1.35	0.98	204	1.57	4.42
Total Physician Services Claims Database (ICD-9 codes)		979	29.94	21.80	5,225	40.16	113.09
Prescription Drug Database							
Cholinesterase Inhibitor							
02232043, 02232044	Aricept	6+	*	*	1,068	8.21	23.12
02242115-02242118, 02245240	Exelon	<6	*	*	63	0.48	1.36
02244298-02244300, 02266717, 02266725, 02266733	Reminyl	<6	*	*	337	2.59	7.29
Total Prescription Drug Database		214	6.54	4.77	1,468	11.28	31.77
Resident Assessment Index – Minimum Data Set (RAI-MDS)							
CPS Scale Score^e^							
2	Mild impairment (MMSE EA 19)	442	13.52	9.84	1118	8.59	24.20
3	Moderate impairment (MMSE EA 15)	441	13.49	9.82	1359	10.44	29.41
4	Moderately severe impairment (MMSE EA 7)	78	2.39	1.74	202	1.55	4.37
5	Severe impairment (MMSE EA 5)	110	3.36	2.45	355	2.73	7.68
6	Very severe impairment (MMSE EA 1)	35	1.07	0.78	91	0.70	1.97
Disease category							
mds_l1r	Alzheimer’s Disease and CPS < 2	<6	*	*	11	0.08	0.24
mds_l1v	Dementia other than Alzheimer’s Disease and CPS < 2	6+	*	*	81	0.62	1.75
Total Resident Assessment Index – Minimum Data Set (RAI-MDS)		1,144	34.98	25.48	3,217	24.72	69.63
	TOTAL four databases	3,270	100.00	72.83	13,012	100.00	281.63

*Not reported due to an insufficient number of cases (i.e., between 1 and 5 cases) or an indeterminate number of cases

^a^Incident number of case defined dementia (N = 3,270)

^b^Population at risk for incident dementia (N = 449,012)

^c^Prevalent number of case defined dementia (N = 13,012)

^d^Population at risk for prevalent dementia (N = 462,024)

^e^Mini-Mental State Examination Equivalent Average (MMSE EA) scores derived from Morris et al. 1994 [50]

Table 8 shows that prevalent cases were most likely to be first identified by a diagnosis in physician services claims (40.16 %). A further 24.72 % were first identified in long-term care, 23.84 % by a diagnosis in hospital, and 11.28 % by a cholinesterase inhibitor prescription. Approximately 70 % of all prevalent cases were first identified with one of five diagnosis codes and other criteria: an ICD-9 diagnosis of ‘other cerebral degenerations’ in physician data (24.63 %), an ICD-10-CA diagnosis of ‘unspecified dementia’ in hospital data (13.35 %), an ICD-9 diagnosis of ‘senile and presenile organic psychotic conditions’ in physician data (13.13 %), and a CPS Scale score indicating moderate (10.44 %) or mild (8.59 %) impairment in long-term care.

As shown in Table 9, 79.72 % (912/1,144) of all incident cases that were first identified in long-term care were identified at the point of admission. The remaining 20.28 % (232/1,144) were admitted to long-term care prior to April 1, 2012 (in some cases by many years) and were not identified as having dementia until 2012/13. Therefore, of all incident cases, 27.89 % (912/3,270) were first identified with dementia at admission to long-term care. On further examination, 63.38 % (578/912) of those first identified at the point of admission were first identified with impairment at the moderate to very severe level (CPS Scale Score ≥ 3) or a disease category of Alzheimer’s disease/other dementia. The remaining 36.62 % (334/912) of individuals first identified at the point of admission were identified with mild impairment (CPS Scale Score of 2). Of all prevalent cases first identified in long-term care, 68.89 % (2,216/3,217) were identified at admission, and 31.11 % (1,101/3,217) were identified 30 days or longer after admission. Therefore, of all prevalent dementia cases, 17.03 % (2,216/13,012) were first identified with dementia at admission to long-term care. Of those prevalent cases first identified at admission, 65.29 % (1,447/2,216) were first identified with impairment at the moderate to very severe level (CPS Scale Score ≥ 3) or a disease category of Alzheimer’s disease/other dementia and 34.70 % (769/2,216) were identified with mild impairment (CPS Scale Score of 2).

**Table 9:** Timing of identification of incident and prevalent dementia in Long-term Care among adults 45 years of age and older, Saskatchewan, by criteria at first identification (April 1, 2012 to March 31, 2013)

Criteria at first identification of dementia	Incident cases (N = 1,144)				Prevalent cases (N = 3,217)			
	First identification at admission^a^		First identification ≥30 days after admission^b^		First identification at admission^b^		First identification ≥30 days after admission^b^	
	n	%	n	%	n	%	n	%
CPS Scale Score^c^								
2 Mild impairment (MMSE EA 19)	334	75.56	108	24.44	769	68.78	349	31.22
3 Moderate impairment (MMSE EA 15)	358	81.17	83	18.83	945	69.53	414	30.46
4 Moderately severe impairment (MMSE EA 7)	70	89.74	8	10.26	145	71.78	57	28.22
5 Severe impairment (MMSE EA 5)	95	86.36	15	13.64	241	67.89	114	32.11
6 Very severe impairment (MMSE EA 1)	28	80.0	7	20.0	52	57.14	39	42.85
Disease category								
Alzheimer disease and CPS <2	<6	*	<6	*	6+	*	<6	*
Dementia other than Alzheimer disease and CPS <2	6+	*	6+	*	6+	*	6+	*
Total	912	79.72	232	20.28	2,216	68.89	1,001	31.11

*Not reported due to an insufficient number of cases (i.e., between 1 and 5) or an indeterminate number of cases

^a^Admission occurred April 1, 2012 to March 31, 2013

^b^Admission occurred prior to April 1, 2012

^c^Mini-Mental State Examination Equivalent Average (MMSE EA) scores derived from Morris et al. 1994 [50]

## Discussion

In the present study, we determined the 2012/13 12-month incidence and prevalence of dementia among adults aged 45 years and older, as identified by a case definition algorithm applied to linked administrative health databases for the province of Saskatchewan (physician services claims, hospital discharge abstracts, prescription drug, and long-term care).

Findings from the present study may be compared with findings from similar registry studies as well as with findings from field studies; however, it should be noted that incidence and prevalence rates from registry studies of dementia are often more comparable to one another than to rates based on field studies. Variations in findings between the present study and previous Canadian registry studies of dementia may be due to a number of factors. First, variations may be attributed to the use of different case definition algorithms, i.e., the use of different age cut-offs, as well as different periods of time and administrative databases. A lower age cutoff, as was used in the present study and in the study by Jacklin and colleagues [43], results in a lower rate of prevalence. Second, a longer observation period such as five years, as was used in most previous Canadian registry studies of dementia [39–42] likely results in a higher prevalence rate than a shorter observation period such as 12 months, as was used in the present study. Finally, employing a greater number of databases, particularly the RAI-MDS (LTC) database as in the present study, likely results in a higher rate than using few databases, as in the study by Jacklin and colleagues [43].

### Incidence and prevalence

The present study determined that the overall incidence rate of dementia among individuals aged 45 years and older was 0.73 % (7.28 per 1,000 PAR). To the best of our knowledge, no previous Canadian study has reported on the incidence of dementia based on RAI-MDS (i.e., long-term care data) linked with physician or hospital administrative data. In the absence of comparable Canadian registry data, the Canadian Study of Health and Aging [20] indicated an overall incidence rate of 20.6 per 1,000 based on a field study of community-dwelling and institutionalized Canadians aged 65 years and older. When focusing solely on incidence among those 65 years and older in the present study, we find a similar rate of 19.1 per 1,000 PAR (3,023/157,907).

For the same 2012/13 period, the overall prevalence rate of dementia among individuals aged 45 years and older in the present study was 2.82 % (28.16 per 1,000 PAR). A previous Canadian registry study of dementia reported a 12-month age-adjusted prevalence rate of 0.56 % among non-First Nations vs. 0.75 % among First Nations of all ages [43]. Other previous Canadian registry studies have reported 5-year prevalence rates ranging from 6.76 % among adults aged 66 and older [42] to rates among adults aged 55 and older ranging from 10.6 % [40] and 10.8 % [41] to 16.6 % [39].

In the present study, the 12-month prevalence rate was 3.9 times the 12-month incidence rate, indicating an average duration of survival from time of identification of 3.9 years. Furthermore, females survived 0.64 years longer on average than males, specifically 4.11 years vs. 3.47 years from time of identification. In comparison, a recent report by WHO and ADI [1] estimated worldwide annual dementia prevalence to be greater than annual incidence by a factor of 4.6, indicating that the average duration of survival, worldwide, was 4.6 years.

### Incidence and prevalence by sex

Within age groups, significant sex differences in age-standardised incidence rates of dementia were apparent only in those 85 and older, with the standardised incidence rate 10 % lower among females than males. Furthermore, significant sex differences in age-standardised prevalence rates were also apparent only in the 85 and older age group, where the standardised prevalence rate was 18 % higher among females than males. Overall, the age-standardised incidence rate was significantly lower among females than males aged 45 years and older by 8 % (7.04 vs. 7.65 per 1,000 PAR), however, the age-standardised prevalence rate was significantly higher among females than males by 9 % (28.92 vs. 26.53 per 1,000 PAR).

To the best of our knowledge, only one previous Canadian registry study based on linked data (without long-term care information), has examined dementia prevalence by sex and age. For all ages 66 years and older, Gill et al. [42] found the prevalence rate to be 23 % higher among females than males. Gill and colleagues [42] reported prevalence rates that were similar in females and males aged 66 to 74 years (2.7 % and 2.6 %, respectively), but slightly higher in females than males aged 75 to 84 years (9.1 % vs. 8.3 %) and 85 years and older (20.3 % vs. 18.3 %). Findings from the Canadian Study of Health and Aging [53], a large field study with community and institutionalized samples combined, also reported prevalence rates that were higher among females than males and that varied with age; the overall prevalence rate among those aged 65 and older was 25 % higher among females than males (86 vs. 69 per 1,000).

According to a WHO and ADI [1] systematic review of 147 field studies published between 1980 and 2004, the overall prevalence was 19 % to 29 % higher among females than males aged 60 and older, with the exception of Asia Pacific and North America. Prince et al. [54] concluded that a higher prevalence rate among females than males, most notably among the oldest-old, suggests that the average duration of survival is lengthier in females than males. Similarly, a US Alzheimer’s Association report based mainly on field study data, concluded that a greater prevalence of dementia among females than males in the oldest age groups indicates that females are likely to live longer with dementia than men [18]. The significantly higher prevalence rate among females than males aged 85 years and older in the current study would seem to support these observations. Longer life expectancy among females than males provides evidence for these findings, with average life expectancy worldwide slightly higher among females (72.7 years) than males (68.1 years) [55].

Thies and Bleiler [18] suggest that negligible sex differences in incidence across all age groups indicate that females are no more likely than males to develop dementia. The current study supports this observation to a degree, as the age-standardised incidence rates did not significantly differ by sex until age 85, at which point the standardised rate was significantly lower among females than males. This finding may be explained by a lower likelihood of dementia in females than males aged 85 and older, lower use of health care services among females than males in this age group, or lower identification of dementia among females than males in this age category by health care professionals. Further examination of these data is necessary to explore sex differences in incidence rates across health care settings.

### Incidence and prevalence by age group

In the present study, the incidence rate escalated by 2.8 to 5.1 times every 10 years, increasing 152-fold between adults aged 45 to 54 and those aged 85 years and older (0.46 vs. 69.73 per 1,000 PAR). Further, the incidence rate was 17.7 times higher among adults 85 years and older than 65 to 74 years (69.73 vs. 3.95 per 1,000 PAR). These findings are consistent with other studies. The CSHA study [20] reported that incidence doubled every five years, with incidence 19.4 times higher among adults 85 years and older than 65 to 70 years (106.5 vs. 5.5 per 1,000). According to a WHO and ADI [1] systematic review of field studies, incidence among adults 60 years and older doubled every 5.9 years, from 3.1 per 1,000 person years among adults 60 to 64 years to 175.0 per 1,000 person years after age 95 years. In the present study, incidence peaked at 6.97 % (69.73 per 1,000 PAR) after age 85 years. Similarly, the WHO and ADI report [1] found that North American dementia incidence peaked between ages 80 and 89.

The 12-month prevalence rate in the present study increased by between 2.6 and 4.6 times every 10 years after age 45 years. Overall, the prevalence rate increased 13.5 times between ages 65 to 74 and 85 and older (16.4 vs. 221.3 per 1,000 PAR), and grew 160-fold between ages 45 to 54 and 85 years and older (1.38 to 221.3 per 1,000 PAR). The single comparable Canadian registry study that examined dementia prevalence by age found an increase of 7.3 times between the age groups 65 to 74 and 85 and older [42]. The findings of the present study are consistent with the CSHA [20] findings that prevalence increased 14.4 times between the age groups 65 to 74 and 85 and older (24 vs. 345 per 1,000). Further, a WHO and ADI [1] systematic review concluded that dementia prevalence doubled with every 5.5 years of age.

A recent review of field and registry studies of early onset dementia (before aged 65 years), conducted in European countries, USA, and Japan, found prevalence rates ranging from 0.38 to 4.2 per 1,000 among individuals ages 20 to 64 years [56]. The prevalence rates among individuals aged 45 to 54 years and 55 to 64 years in the present study are higher in comparison at 1.38 and 6.11 per 1,000 PAR, respectively. This difference may be due partly to the higher age cutoff in the present study at 45 years, compared with a lower age cutoff of 20 years in the review. Although early onset dementia is less common than dementia in adults over aged 65 years, it is important to consider the different nature of the impact of dementia on these individuals and their families, who are more likely to be employed in the workforce, under greater financial pressures, with younger families and perhaps frail parents [21].

### Incidence and prevalence by diagnosis code and other criteria

In the present study, incident cases were first identified more often in long-term care (34.98 %) than other settings (29.94 % physician services claims; 28.53 % hospital; 6.54 % cholinesterase inhibitor prescription). Of all incident cases, 27.89 % were first identified at the point of admission to long-term care. These findings indicate that approximately one in four individuals with incident dementia were admitted to long-term care before a formal diagnosis of dementia was recorded in physician or hospital data. These findings also underscore the issue of underdiagnosis, attributable to many factors internal and external to the health system [10, 11, 14].

Prevalent cases in the present study were more likely to be first identified in physician services claims (40.16 %) than other settings (24.72 long-term care; 23.84 % hospital; 11.28 % cholinesterase inhibitor prescription). Diagnoses in physician data were non-specific as a function of the four ICD-9 codes available (290, 294, 331, and 797); 24.63 % of all prevalent cases were first identified with a formal diagnosis of ‘other cerebral degenerations’ and 13.13 % with a diagnosis of ‘senile and presenile organic psychotic conditions’. A further 13.35 % of all prevalent cases were first identified with a diagnosis of ‘unspecified dementia’ in hospital data. Given the large number of diagnosis codes available in hospital (25 vs. one code in physician data), this finding suggests that the tendency among physicians to choose non-specific diagnosis codes regardless of the number of codes available may reflect diagnostic uncertainty or a large proportion of mixed pathologies. Alzheimer disease (AD) is the most common type of dementia, and mixed pathologies in dementia are far more common than pure pathologies [1]. A study of 1,050 autopsy dementia cases found that the large majority (62.9 %) were likely Alzheimer Disease, and a minority were non-AD, specifically 10.4 % were nonspecific degenerative dementia, 10.0 % vascular dementia, 9.5 % Parkinson disease with dementia, and 1.5 % mixed dementia [57]. Further, fewer than half (43 %) were likely pure Alzheimer’s disease.

### Limitations

This study may have resulted in either an over- or an under-estimation of incidence and prevalence rates. First, given that dementia is significantly underdiagnosed in the health care system, studies based on administrative data tend to underestimate the number of individuals with dementia, particularly among older age groups [56]. In comparison, field studies tend to have greater sensitivity for identifying cases and therefore generally report higher rates than registry studies [56]. In the present study, underestimation also may have resulted from not including home care data in our analysis (as it was not available). Furthermore, physician services claims allow a maximum of one diagnosis code per claim, which may hinder physicians from recording a dementia-related diagnosis for patients with co-morbid conditions or conditions that are less challenging to diagnose and may in turn result in underestimation in physician data. In addition, reluctance among individuals to seek help from health care professionals for issues related to cognitive function contributes to the likelihood that physician data underestimate the true incidence and prevalence of dementia. Second, overestimation of incidence and prevalence rates may have occurred as a result of several factors. For instance, the case definition algorithm developed for this study required only one diagnosis or other criterion of dementia. However, this algorithm is comparable to other Canadian studies of dementia epidemiology that also employed a minimum of one diagnosis in physician or hospital data. It is also possible that individuals who were not identified in physician data (perhaps due to the fact that one diagnosis code per claim is allowed) were later identified with dementia in the hospital discharge abstract database which permits up to 25 diagnoses per record, resulting in overestimation in hospital data. Overestimation may also have resulted from identifying individuals with incident dementia when they had fewer than five years of uninterrupted health insurance prior to their index date (i.e., gaps of no more than 3 consecutive days). Lastly, the Cognitive Performance Scale (CPS) score used in our algorithm, derived from the provincial RAI-MDS (long-term care) database, focuses primarily on cognitive function and therefore may overestimate incidence and prevalence of dementia (since a diagnosis requires both functional and cognitive impairment).

## Conclusions

In the present study, we linked together 10 provincial-level databases, including four administrative health databases (physician, hospital, prescription drug, and long-term care), to determine the incidence and prevalence of dementia among adults aged 45 years and older across multiple settings. This study found that for every 10 years of age after age 45 years, the incidence rate increased by 2.8 to 5.1 times and the prevalence rate increased by 2.6 to 4.6 times. The findings indicated an average duration of survival from time of diagnosis of 3.9 years, with females surviving an average of 0.64 years longer than males. After age standardisation, the incidence rate was significantly lower but the prevalence rate was significantly higher overall among females than males, due largely to significant sex differences in the oldest age group. Among females compared to males aged 85 and older, the lower incidence rate but higher prevalence rate of dementia suggest a lower likelihood of dementia or perhaps lower formal recognition, but longer disease duration, among females than males in this age group.

We also found that approximately one-quarter of all incident cases were admitted to long-term care before a diagnosis was formally recorded in physician or hospital data, and 63.38 % (578/912) of these cases were identified at admission with impairment at the moderate to very severe level (CPS Scale Score ≥ 3) or a disease category of Alzheimer’s disease/other dementia. We suggest that the maximum limit of one diagnosis code per service claim hinders physicians from formally recording a diagnosis for a greater number of individuals with dementia, and therefore recommend that the number of diagnosis codes allowed per physician services claim be increased. Linking multiple sources of registry data contributes to our understanding of the epidemiology of dementia across multiple segments of the population, inclusive of individuals residing in long-term care. Future research that employs linked administrative would ideally incorporate Home Care data to determine whether this dataset contains additional cases of dementia.

## Additional file

Additional file 1:
**Diagnosis codes and other criteria employed to identify dementia cases in administrative health databases, by Canadian study.**

## Abbreviations

ADI: Alzheimer’s Disease International
CSHA: Canadian Study of Health and Aging
DIN: Drug identification number
HQC: Health Quality Council
MCHP: Manitoba Centre for Health Policy
WHO: World Health Organization
