# Supplementary material for: Incidence and prevalence of dementia in linked administrative health data in Saskatchewan, Canada: a retrospective cohort study
Source: BMC Geriatr. 2015 Jul 3;15:73. doi: 10.1186/s12877-015-0075-3 (PMC4489119; doi:10.1186/s12877-015-0075-3)
Supplement: Additional file 1: — Diagnosis codes and other criteria employed to identify dementia cases in administrative health databases, by Canadian study. [file 12877_2015_75_MOESM1_ESM.pdf]

Additional file 1: **Diagnosis codes and other criteria employed to identify dementia cases in administrative health databases, by Canadian study**[a]

Diagnosis codes	Publication/Study							
	Present Study	Manitoba Centre for Health Policy [38]	Chartier et al. [39]	Martens et al. [40]	Fransoo et al. [41]	Gill et al. [42]	Jacklin et al. [43]	Jacklin and Walker [44]
**Hospital Discharge Abstract Database ICD-9 (1996-2001)**								
290.0 Senile dementia, simple type	Y	Y	Y	Y	Y	Y		Y
290.1 Presenile dementia	Y	Y	Y	Y	Y	Y		Y
290.2 Senile dementia, depressed or paranoid type	Y	Y	Y	Y	Y	Y		Y
290.3 Senile dementia with acute confusional state	Y	Y	Y	Y	Y	Y		Y
290.4 Arteriosclerotic dementia	Y	Y	Y	Y	Y	Y		Y
290.8 Other senile and presenile organic psychotic conditions	Y	Y	Y	Y	Y	Y		Y
290.9 Unspecified senile and presenile organic psychotic conditions	Y	Y	Y	Y	Y	Y		Y
291.1 Korsakov's psychosis, alcoholic	Y	Y	Y	Y	Y			
291.2 Other alcoholic dementia	Y	Y	Y	Y	Y			
292.8 Other drug psychoses	Y	Y	Y	Y	Y			
294.0 Korsakov's psychosis or syndrome (nonalcoholic)	Y	Y	Y	Y	Y			Y
294.1 Dementia in conditions classified elsewhere	Y	Y	Y	Y	Y			Y

Diagnosis codes	Publication/Study							
	Present Study	Manitoba Centre for Health Policy [38]	Chartier et al. [39]	Martens et al. [40]	Fransoo et al. [41]	Gill et al. [42]	Jacklin et al. [43]	Jacklin and Walker [44]
294.8 Other organic psychotic conditions (chronic)	Y	Y	Y	Y	Y			Y
294.9 Unspecified other organic psychotic conditions (chronic)	Y	Y	Y	Y	Y			Y
331.0 Alzheimer's disease	Y	Y	Y	Y	Y	Y		Y
331.1 Pick's disease	Y	Y	Y	Y	Y	Y		Y
331.2 Senile degeneration of brain	Y	Y	Y	Y	Y	Y		Y
331.3 Communicating hydrocephalus	Y	Y	Y	Y	Y	Y		Y
331.4 Obstructive hydrocephalus	Y	Y	Y	Y	Y	Y		Y
331.5 Jakob-Creutzfedlt disease	Y	Y	Y	Y	Y	Y		Y
331.6 Progressive multifocal leucoencephalopathy	Y	Y	Y	Y	Y	Y		Y
331.7 Cerebral degenderation in other diseases classified elsewhere	Y	Y	Y	Y	Y	Y		Y
331.8 Other cerebral deeneration	Y	Y	Y	Y	Y	Y		Y
331.9 Unspecified	Y	Y	Y	Y	Y	Y		Y
797 Senility without mention of psychosis	Y	Y	Y	Y	Y	Y		Y
**Hospital Discharge Abstract Database ICD-10-CA (2001-2013)**								
F00.0 Dementia in Alzheimer's Disease with early onset	Y	Y	Y	Y	Y	Y		Y
F00.1 Dementia in Alzheimer's Disease with late onset	Y	Y	Y	Y	Y	Y		Y

Diagnosis codes	Publication/Study							
	Present Study	Manitoba Centre for Health Policy [38]	Chartier et al. [39]	Martens et al. [40]	Fransoo et al. [41]	Gill et al. [42]	Jacklin et al. [43]	Jacklin and Walker [44]
F00.2 Dementia in Alzheimer's Disease, atypical or mixed type	Y	Y	Y	Y	Y	Y		Y
F00.9 Dementia in Alzheimer's Disease, unspecified	Y	Y	Y	Y	Y	Y		Y
F01.0 Vascular dementia of acute onset	Y	Y	Y	Y	Y	Y		Y
F01.1 Multifarct dementia	Y	Y	Y	Y	Y	Y		Y
F01.2 Subcortical vascular dementia	Y	Y	Y	Y	Y	Y		Y
F01.3 Mixed cortical and subcortical vascular dementia	Y	Y	Y	Y	Y	Y		Y
F01.8 Other vascular dementia	Y	Y	Y	Y	Y	Y		Y
F01.9 Vascular dementia, unspecified	Y	Y	Y	Y	Y	Y		Y
F02.0 Dementia in Pick's disease	Y	Y	Y	Y	Y	Y		Y
F02.1 Dementia in Creutzfeldt-Jakob disease	Y	Y	Y	Y	Y	Y		Y
F02.2 Dementia in Huntington's disease	Y	Y	Y	Y	Y	Y		Y
F02.3 Dementia in Parkinson's disease	Y	Y	Y	Y	Y	Y		Y
F02.4 Dementia in human immunodeficiency virus HIV disease	Y	Y	Y	Y	Y	Y		Y
F02.8 Dementia in other specified diseases classified elsewhere	Y	Y	Y	Y	Y	Y		Y
F03 Unspecified dementia	Y	Y	Y	Y	Y	Y		Y
F04 Organic amnesic syndrome, not induced by alcohol and other psychoactive substances	Y	Y	Y	Y	Y			

Diagnosis codes	Publication/Study							
	Present Study	Manitoba Centre for Health Policy [38]	Chartier et al. [39]	Martens et al. [40]	Fransoo et al. [41]	Gill et al. [42]	Jacklin et al. [43]	Jacklin and Walker [44]
F05.1 Delirium superimposed on dementia	Y	Y	Y	Y	Y	Y		
F06.8 Other specified mental disorders due to brain damage and dysfunction and to physical disease	Y	Y	Y	Y	Y	Y		
F06.9 Unspecified mental disorder due to brain damage and dysfunction and to physical disease	Y	Y	Y	Y	Y	Y		
F09 Unspecified organic or symptomatic mental disorder	Y	Y	Y	Y	Y	Y		
F10.6 Mental and behavioural disorders due to use of alcohol, amnesic syndrome	Y			Y	Y			
F10.7 Mental and behavioural disorders due to use of alcohol, residual and late-onset psychotic disorder	Y	Y	Y	Y	Y			
F18.6 Mental and behavioural disorders due to use of volatile solvents, amnesic syndrome	Y			Y	Y			
F18.7 Mental and behavioural disorders due to use of volatile solvents, residual and late-onset psychotic disorder	Y	Y	Y	Y	Y			
F19.6 Mental and behavioural disorders due to multiple drug use and use of other psychoactive substances, amnesic syndrome	Y			Y	Y			

Diagnosis codes	Publication/Study							
	Present Study	Manitoba Centre for Health Policy [38]	Chartier et al. [39]	Martens et al. [40]	Fransoo et al. [41]	Gill et al. [42]	Jacklin et al. [43]	Jacklin and Walker [44]
F19.7 Mental and behavioural disorders due to multiple drug use and use of psychoactive substances, residual and late-onset psychotic disorder	Y	Y	Y	Y	Y			
G30.0 Alzheimer's disease with early onset	Y	Y	Y	Y	Y	Y		Y
G30.1 Alzheimer's disease with late onset	Y	Y	Y	Y	Y	Y		Y
G30.8 Other Alzheimer's disease	Y	Y	Y	Y	Y	Y		Y
G30.9 Alzheimer's disease, unspecified	Y	Y	Y	Y	Y	Y		Y
G31.0 Circumscribed brain atrophy	Y	Y	Y	Y	Y	Y		
G31.1 Senile degeneration of brain, not elsewhere classified	Y	Y	Y	Y	Y	Y		
G91.0 Communicating hydrocephalus	Y	Y	Y	Y	Y			
G91.2 Normal-pressure hydrocephalus	Y	Y	Y	Y	Y			
R54 Senility	Y	Y	Y	Y	Y	Y		
**Physician Services Claims Database & Physician Characteristics Database ICD-9 (2001-2013)**								
290 Senile and presenile organic psychotic conditions	Y	Y	Y	Y	Y	Y	Y	Y
294 Other organic psychotic conditions chronic	Y	Y	Y	Y	Y			Y
331 Other cerebral degenerations	Y	Y	Y	Y	Y	Y	Y	Y
797 Senility without mention of psychosis	Y	Y	Y	Y	Y	Y		

Diagnosis codes	Publication/Study							
	Present Study	Manitoba Centre for Health Policy [38]	Chartier et al. [39]	Martens et al. [40]	Fransoo et al. [41]	Gill et al. [42]	Jacklin et al. [43]	Jacklin and Walker [44]
**Prescription Drug Database (2001-2013)**								
Aricept (02232043, 02232011)	Y					Y		
Exelon (0224215-0224218, 02245240)	Y					Y		
Reminyl (02244298-02244300, 02266717, 02266725, 02266733)	Y					Y		
**Resident Assessment Index – Minimum Data Set (RAI-MDS) (2004-2013)**								
Cognitive Performance Scale Score $\geq$ 2	Y							
Diagnosis of Alzheimer's Disease	Y							
Diagnosis of Dementia other than Alzheimer's Disease	Y							

[a] All diagnosis codes and other criteria that were used to identify dementia cases in the present study are listed in this table. However, some of the cited studies used additional codes that are not listed in this table.

Note. In line with MCHP recommendations, and in contrast to earlier Canadian studies of dementia in hospital data 2001 and onward [40,41] the present study excluded most ICD-10-CA codes of 'mental and behavioural disorders' due to the use of alcohol, illicit drugs, sedatives, stimulants, tobacco, volatile solvents, and other multiple drug use combined with psychoactive substances (exceptions noted above) [i.e., F10.5, F11.0, F11.3-11.6, F11.8, F11.9, F12.0, F12.3-12.6, F12.8, F12.9, F13.0, F13.3-F13.6, F13.8, F13.9, F14.0, F14.3-14.6, F14.9, F15.0, F15.3-15.6, F15.8, F15.9, F16.0, F16.3-16.6, F16.8, F16.9, F17.0, F17.3-F17.9, F18.0, F18.3-F18.5, F18.8, F18.9, F19.0, F19.3-19.5, F19.8, F19.9]. In contrast to MCHP recommendations and earlier studies [40,41], the present study excluded ICD-10-CA codes of 'mental and behavioural disorders residual and late-onset psychotic disorder', due to use of illicit drugs, sedatives, and stimulants (i.e., F11.7, F12.7, F13.7. F14.7. F15.7. F16.7). Further, in contrast to MCHP recommendations and earlier studies [40,41], the present study excluded certain ICD-10-CA codes of 'degenerative diseases of nervous system' (G31.9, G32.8), certain 'hydrocephalus' codes (G91.1, G91.3, G91.8, G91.9), 'Reye's syndrome' (G93.7), and all 'other disorders of brain in diseases classified elsewhere' (G94.0, G94.1, G94.2, G94.8). The present study also excluded ICD-10-CA codes of 'organic dissociative disorder (F06.5) and 'organic emotionally labile disorder' (F06.6), in contrast to MCHP recommendations and earlier studies [40,41,43].
